# Determinants of Nocturnal Cardiovascular Variability and Heart Rate Arousal Response in Restless Legs Syndrome (RLS)/Periodic Limb Movements (PLMS)

**DOI:** 10.3390/jcm8101619

**Published:** 2019-10-04

**Authors:** Emilia Sforza, Frédéric Roche, Vincent Pichot

**Affiliations:** EA SNA-EPIS Service de Physiologie Clinique et de l’Exercice (Pole Hospitalier NOL), CHU de Saint-Étienne, Faculté de Médecine Jacques Lisfranc, Université Jean Monnet, Université de Lyon, 42055 Saint-Étienne, France; emilia.sforza@gmail.com (E.S.); frederic.roche@univ-st-etienne.fr (F.R.)

**Keywords:** restless legs, periodic legs movement, autonomic system, autonomic system, heart rate arousal response, electromyographic activity, sympathetic activity

## Abstract

Recent studies have suggested that restless legs syndrome is associated with an increased prevalence of cardiovascular diseases mediated by sympathetic activation occurring during periodic limb movements. The aim of this study was to establish which factors affect the degree of sympathetic activation during the basal condition and during periodic limb movements that may contribute to increased vascular risk. Fifty untreated restless legs syndrome patients aged 62.6 ± 11.1 y, free of cardiovascular diseases, were examined. Heart rate variability was calculated during wakefulness and all sleep stages, during periods with and without periodic limb movements. Heart rate changes before and after periodic limb movement onset were analyzed to assess the arousal response to periodic limb movements. Both analyses took into account the effects of age, gender, periodic limb movement duration, periodic limb movement index, periodic limb movement interval and periodicity, and magnitude of muscular activity (electromyogram power). Compared to periods without periodic limb movements, a significant increase in sympathetic activity occurred in periods with periodic limb movements, independent of age, sex and periodic limb movement characteristics. Data obtained from the cardiac arousal response to periodic limb movements showed that electromyogram power is the factor affecting sympathetic tonus. These results suggest that other factors, such as electromyogram power and individual susceptibility, should be considered in the assessment of the vascular risk related to restless legs syndrome.

## 1. Introduction

Restless legs syndrome (RLS) is a common sensorimotor neurological disease with a prevalence of up to 11% in the general population [1,2,3]; it is more frequent in females [4] and increases in prevalence with age [3]. Diagnosis is made using clinical criteria: (i) urgency to move the legs frequently associated with unpleasant leg sensations; (ii) worsening of the symptoms at rest, sitting or lying down; (iii) partial and temporary relief by activity; and (iv) worsening later in the day or night. In about 80–90% of subjects, periodic limb movements (PLMS), which are leg movements lasting 0.5–10 s, occurring in a series of at least four movements with at least 8 µV in amplitude and an intermovement interval between 5 and 90 s, are present during sleep.

In recent years, clinical [5,6,7,8] and epidemiological studies [9,10] have established an association between RLS-PLMS and increased incidences of hypertension, cardiovascular diseases, and mortality after adjustment for cardiovascular risk factors, with a greater risk among those who have more frequent symptoms [11] and a RLS diagnosis for more than 3 years [3,9]. However, other studies [12,13,14] have failed to find this association, and there is a persistent controversy [15,16] over whether there is a real link between RLS/PLMS and cardiovascular risk.

We know that RLS/PLMS may induce a nocturnal increase in cardiac sympathetic activity [17] and blood pressure [18] as a consequence of two factors: sleep fragmentation and PLMS itself. Sleep fragmentation does not seem to play a key role in vascular risk, since not all PLMS are associated with cortical arousal [17]. Palma et al. [19] examined 13 middle-aged RLS patients and 13 matched controls, and they did not find differences in heart rate variability (HRV) measurements between wakefulness and periods of sleep without PLMS, suggesting a basal normal tone in middle-aged PLMS patients. Additionally, it has been shown that the sympatho-vagal balance is significantly increased during periods with PLMS compared to periods without PLMS [20]. Thus, the cardiac and vascular changes around PLMS seem to be key factors explaining the changes in the autonomic nervous system (ANS) in RLS/PLMS patients. An interesting point is that, except for age, sex [21] and duration of illness [11], neither frequency nor the PLMS duration seems to significantly affect the vascular response. It must be noted that these results were obtained in small samples without data on the presence of other health problems and on vascular medications that may have affected the validity of the results.

The objectives of the current study were two-fold: (i) to examine, in a group of untreated RLS/PLMS patients, if autonomic nervous changes occurred not only during periods of sleep with PLMS but also during wakefulness and periods without PLMS in order to assess if a persistent sympathetic hyperactivity is present in both conditions; and (ii) to assess which factors among age, gender and intrinsic PLMS characteristics, including frequency, duration, interval, periodicity and strength of muscular leg activity, contribute most to cardiac responses to PLMS, and the consequent vascular risk.

## 2. Experimental Section

### 2.1. Subjects

Over a 3-year period, all patients examined for insomnia and/or probable RLS completed a face-to-face clinical interview, neurological examination, standardized questionnaires for RLS and insomnia, and nocturnal polysomnography. The patients with a diagnosis of primary RLS met the classical standard criteria for the diagnosis: urgency to move the legs frequently associated with unpleasant leg sensations; worsening of the symptoms at rest, sitting or lying down; partial and temporary relief by activity; and worsening later in the day or night. Exclusion criteria were the following: previous diagnosis of RLS currently treated; any medication (e.g., dopamine agonists, benzodiazepines, antidepressants) that may influence the occurrence of PLMS; coexistent diseases such as diabetes type 1, renal dysfunction; neurological disease treated by dopamine or dopamine-agonists; psychiatric disease treated by medication inducing RLS/PLMS; arrhythmias or absence of sinus rhythm; current use of drugs that have known autonomic or cardiac effects; untreated hypertension; and previous or current diagnosis of obstructive sleep apnea.

The University Hospital and the local Ethics Committee (CCPRB Rhone-Alpes Loire) approved the study. All patients gave their written consent prior to participation in the study.

### 2.2. Clinical Assessment

Clinical evaluation was undertaken by a standard interview, which included history of cardiac and cerebrovascular disease, hypertension, diabetes, and respiratory, neurological and psychiatric disorders. Body mass index (BMI), calculated as weight/height squared (kg/m^2^), was measured. 

### 2.3. At-Home Polysomnography Recording

In all subjects, an ambulatory polysomnography was performed including conventional four electroencephalographic (EEG) tracings, right and left electrooculogram (EOG), chin and bilateral anterior tibialis electromyograms (EMG), electrocardiogram (ECG), nasal pressure, respiratory effort, body position, and oxygen saturation (SaO2) measured by pulse oximetry. All signals were recorded at 250 Hz. Sleep stages of 30 s epochs and respiratory events were manually scored according to AASM scoring rules [22,23]. PLMS were defined as at least four consecutives limb movements with a duration of 0.5–10 s with an intermovement interval between 5–90 s [24]. The PLM index (number/hour), mean duration, and mean interval and periodicity [25,26] were also defined.

### 2.4. HRV Analysis

The raw data for wakefulness, sleep, sleep stages, all scored events including PLMS, bilateral EMG, and ECG traces were extracted from Somnologica Studio® (Version 5.1, Embla, Broomfield, CO, US) using the built-in export data tool. Then, all calculations were performed with in-house software, including HRV analysis [27], developed with Matlab 9.0.0 R2016a (The Mathworks Inc. Natick, MA, USA).

Each R peak was detected on the ECG to provide the R–R interval series. The missing beats, isolated extrasystoles, and artefacts were corrected using a spline cubic interpolation, as suggested in the HRV guidelines [28]. Epochs with body movement or successive artifacts were discarded from analyses.

The standard time and frequency domain indices of HRV were calculated on 5 min epochs according to standard criteria [28]. In the time domain, the mean heart rate (HR), the standard deviation of R–R (SDNN), the root mean square of successive R–R differences (RMSSD), and the percentage of successive R–R that differed by more than 50 ms (pNN50) were calculated. Frequency domain measures based on the fast-Fourier transform algorithm were applied to the regularly resampled R–R signal at 4 Hz. Spectral power was calculated for standard frequency bands: total power (Ptot: 0–0.4 Hz), very low frequency (VLF: 0–0.04 Hz), low frequency (LF: 0.04–0.15 Hz), and high frequency (HF: 0.15–0.4 Hz), as well as the LF/HF ratio.

The SDNN and Ptot indicate the global ANS status. The VLF is notably an index of the regulation of the renin–angiotensin system, thermoregulation, and parasympathetic activity. The pNN50, rMSSD and HF represent the vagal activity. LF contains both sympathetic and parasympathetic activities. The LF/HF ratio represents an estimation of the sympatho-vagal balance [27,28].

The HRV indices were calculated in two different ways:

(a) During quiet wakefulness before sleep and during a stable sleep epoch; that is, a 5 min period during which the sleep stage did not change and without arousal or PLMS. The analysis during waking may suggest the presence of a basal sympathetic hyperactivity as a consequence of chronic PLMS activation during sleep.
(b) The same analysis was performed during stable sleep periods with PLMS, considering the effect of age, sex, PLMS duration, interval, periodicity, density and EMG activity, in order to assess the factor that might play a key role in the increase of sympathetic activity.

The considered sleep stages were light sleep (stage 1–2), slow wave sleep (stage 3), and rapid eye movement (REM) sleep.

### 2.5. HR Response to Leg Movements 

The R signal was converted from ms to beats/min to obtain an HR signal. Then, all 30 s HR sequences starting from −10 s before the onset of leg movements and ending at +20 s afterward were selected if no additional events were scored in the 20 s period surrounding the considered leg movement. From these sequences, the change in HR was calculated as the difference between each HR value and the baseline was defined as the mean HR from −10 s to −6 s before leg movement. Additionally, cardiac activation was calculated as the difference between the maximum HR value during tachycardia and baseline. The individual HR responses were averaged for light, slow wave, REM, and non-REM sleep.

### 2.6. Analysis of Leg Muscular Activity (EMG)

Bilateral electromyographic leg signals were passed through a bandpass Butterworth filter with low and high passes set to 20 Hz and 225 Hz, respectively [29]. Then, the resulting signals were squared to obtain the EMG power. For the analysis, the EMG derivation (left or right), which permitted the diagnosis of the leg movement, was considered.

As for the HR signal, all 30 s EMG sequences starting from −10 s before the onset of leg movements and ending at +20 s afterward were selected and averaged, with a sample rate of 128 Hz. From these sequences, the total power of the EMG (PEMG) was calculated as the area of the curve, from the beginning and the end of the leg movement. 

The individual HR responses were averaged for light, slow wave, REM, and non-REM sleep. 

To assess which factors most affected the arousal cardiac response to PLMS, we considered the effect of age, sex, duration and interval between PLMS, periodicity, and the degree of EMG activity. 

### 2.7. Statistical Analyses

Data were reported as means ± SD for continuous variables and counts and percentages for categorical variables. The thresholds to create age, PLMS index and duration subgroups (age, PLM index, PLMS duration) were calculated as the median of the groups to ensure similar group sizes.

For statistical analyses, the EMG power index and all HRV indices except HR were log-transformed to reach a normal distribution. Differences between subgroups for polysomnographic characteristics, PLMS characteristics, HRV indices, across sleep stages, and evolution of HR and EMG around leg movements were assessed using a t-test. An analysis of variance (ANOVA) was utilized to compare HRV indices between wakefulness and sleep stages, and to compare the baseline HR values prior to the onset of leg movements to the HR following the onset of leg movements. 

Data were analyzed using Staview 5.0 (SAS Institute Inc. Cary, NC, USA) and Matlab Statistics and Machine learning Toolbox 10.2. All reported p-values were two-tailed, with the threshold of statistical significance set at *p* < 0.05.

## 3. Results

### 3.1. Clinical Data and Sleep Parameters

From the original sample of 80 subjects, 50 untreated patients met all of the inclusion criteria. Table 1 reports the nocturnal polysomnographic data in the total group and in each sex. Fifty patients aged 62.6 ± 11.1 y, 22 females and 28 men with a mean BMI of 25.9 ± 3.3 kg/m^2^, were examined. The mean onset of RLS symptoms was 10.9 ± 4.1 y, and their average IRLS rating scale score [30] was 24 ± 4. Polysomnography revealed a decrease in total sleep time, sleep efficiency, and slow wave sleep, and an increase in wakefulness and sleep latency. The mean PLMS index was 45.7 ± 24.7 (ranging from 8.0 to 132.2 n/h) and the mean PLMS duration was 2.8 ± 1.0 s. There were no differences between men and women. Their mean apnea–hypopneas index was 3.9 ± 3.0, allowing exclusion of obstructive sleep apnea.

### 3.2. Muscular EMG Power 

**Figure 1:**
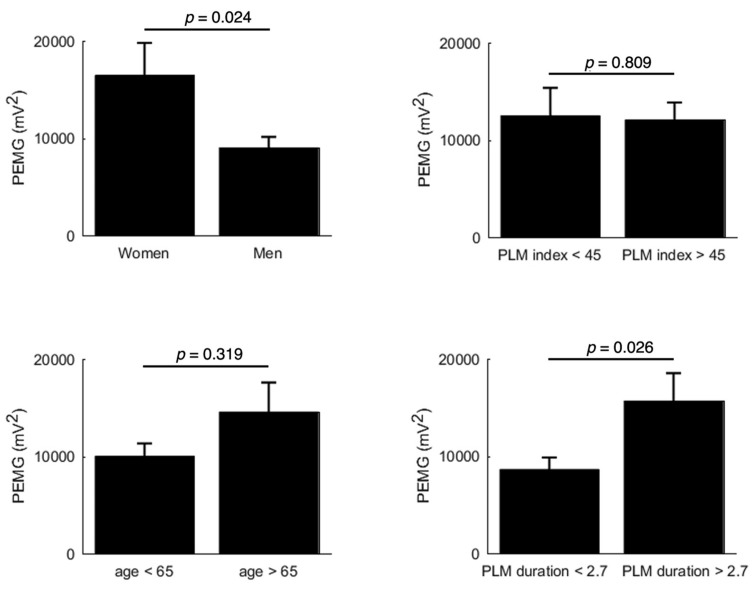
Electromyogram (EMG) power according to age, gender, PLM index, and PLMS duration. Data are presented as mean ± SD but were log-transformed to perform the statistical analyses.

Figure 1 reports the power of EMG activity according to age, sex, PLMS index, and duration. As illustrated, the power of EMG activity differed significantly according to PLMS duration (*p* < 0.03) and gender (*p* < 0.03), without having an effect on the PLMS index (*p* = 0.81) and age (*p* = 0.32).

### 3.3. HRV Analysis During Sleep

Table 2 shows the time and frequency domain data of HRV during wakefulness and each sleep stage, for periods with and without PLMS. Comparison between wakefulness and periods without PLMS showed an expected significant decline in HR, SDNN, VLF, LF and LF/HF during stage 2 and SWS, reflecting physiological changes in autonomic activity toward vagal hypertonus during sleep. When we considered periods with PLMS compared to periods without PLMS, except for the HF values, there was a strong significant increase in VLF, LF, and the LF/HF ratio (*p* < 0.001) in all non-REM sleep stages, without a sympathetic increase in REM sleep.

In order to assess which factor may affect the degree of HRV changes during the periods with PLMS, Table 3 and Table 4 report the possible effects of age, sex, and PLMS duration and index on the HRV variables at each sleep stage. Considering gender and age, while no significant difference was found for sex, older patients had a significant (*p* < 0.05) decrease in LF power in light sleep, without any significant changes in HF. Considering the PLMS characteristics (Table 4), there was an increase (*p* < 0.05) in SDNN, Ptot, VLF and LF when the PLMS mean duration was longer. No effect was found when the PLMS periodicity (PI) and interval (IMI) were considered.

### 3.4. Autonomic Response to PLMS

**Figure 2:**
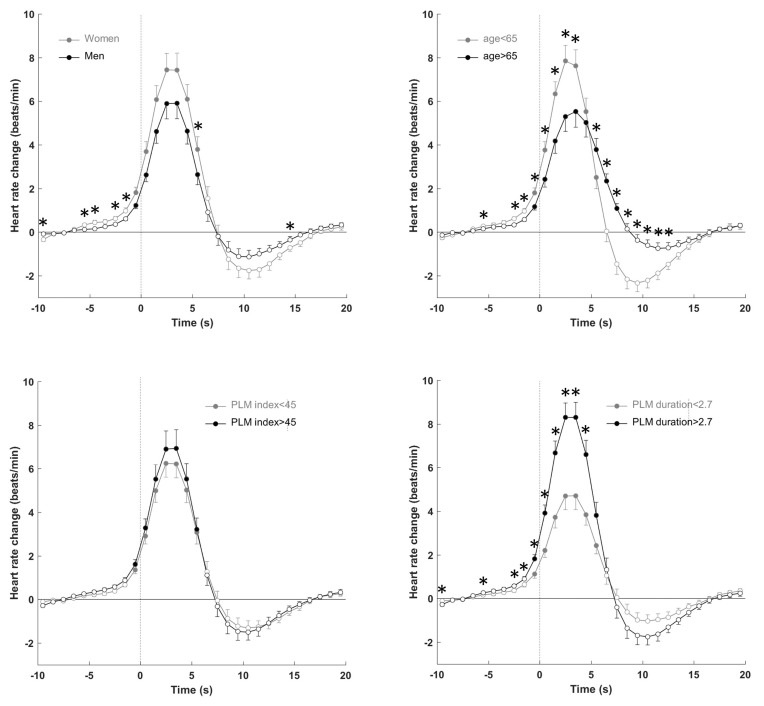
Heart rate response to leg movements during stage 2 according to sex, age, PLMS index, and PLMS duration. Data are presented as means ± SEM. The dashed line represents the leg movement onset. * *p* < 0.05 between sex, age, PLM index and PLMS duration. Filled circles indicate *p* < 0.05 from baseline.

**Figure 3:**
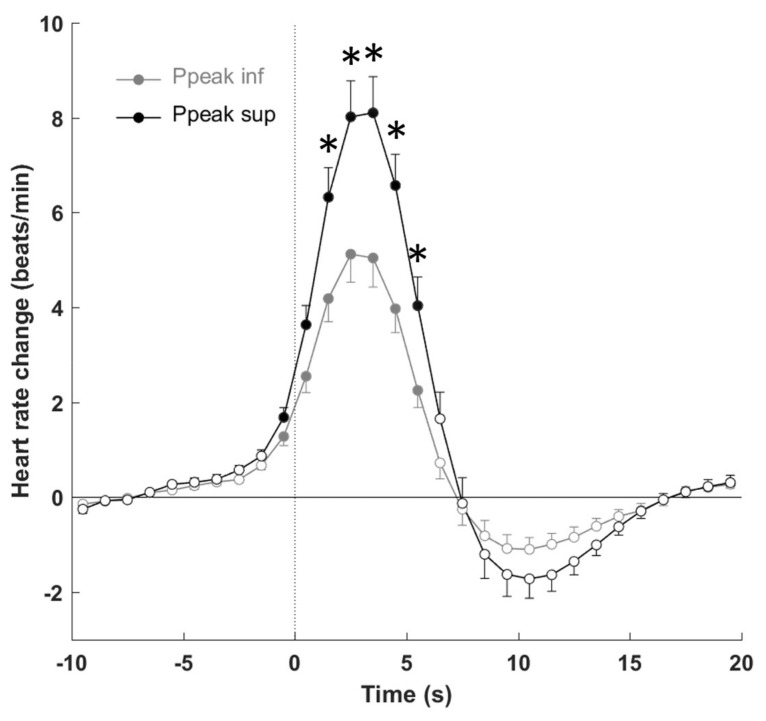
Heart rate response to leg movements measured during stage 2 depending on electromyogram (EMG) power. Ppeak sup and Ppeak inf are the responses for subjects presenting a Ppeak value higher and lower than the median value, respectively. Data are presented as means ± SEM. The dashed line represents leg movement onset. * *p* < 0.05 between Ppeak inf and Ppeak sup. Filled circles indicate *p* < 0.05 from baseline.

Figure 2 reports the evolution over time of R–R changes associated with PLMS according to gender, age, PLM index, PLMS duration, and degree of muscular power. Overall, R–R interval shortening indicating tachycardia started to fall one to two intervals before the onset of the event, peaking during the following intervals from 5–10, with a consequent stabilization in the post-event period. When the magnitude of the PLMS-related HR response was considered, no significant differences were found when the effects of gender and PLMS index were examined. Older patients showed a significant reduction (*p* < 0.05) in the degree of tachycardia before and after the PLMS onset and bradycardia in the post-arousal period. PLMS with a longer duration had a greater tachycardia (*p* < 0.05) in the 5 s after the onset of PLMS, without differences in the post-bradycardia period. Figure 3 shows the more significant sympathetic activation (*p* < 0.01) when the EMG peak was high, without differences in the post-event period. 

## 4. Discussion

To the best of our knowledge, the present study is the first to examine, in a relatively large group of untreated RLS patients free of vascular diseases, the factors affecting the cardiac autonomic system during sleep and the pattern of cardiac response to PLMS, both implicated in the possible association between RLS-PLMS and cardiovascular risk. The first finding of this study is the analysis between wakefulness and periods of non-REM sleep without PLMS, showing a significant reduction of HR and LF/HF power, which probably reflects the physiological decrease in sympathetic activity during sleep. The second finding is that in periods with PLMS, there is strong and significant increase in sympathetic tonus that occurs independently of age, gender, and PLMS severity, duration and periodicity. Finally, when we considered the HR response to PLMS, again neither sex and age nor PLMS density and duration strongly affected the arousal response of tachycardia–bradycardia, with the power of EMG activity being the strongest factor affecting an increase in sympathetic tone. Overall, these data might suggest a role of other mechanisms in RLS cardiovascular risk.

The first interesting finding is that despite the well-known sympathetic hyperactivity associated with PLMS, the HRV analysis during wakefulness and periods without PLMS did not show significant dysfunction of the vagal–sympathetic balance. Despite the lack of a control group in our study, we can hypothesize that the basal autonomic tone in our patients did not reveal a sympathetic hyperactivity, with the changes from wakefulness to stable sleep just reflecting the physiological sleep adaptation of the ANS [31]. Although speculative, our results are in line with previous studies [19,32] that argue against the role of a basal autonomic dysfunction in RLS vascular risk. If so, we can explain the results of Winkelmann et al. [9], Högl et al. [33] and Rothdach et al. [34], stressing the lack of relationship between hypertension and RLS, with the controversial results probably dependent on the inclusions of patients treated for cardiac and vascular diseases [35,36] or having secondary RLS [14].

The second aim of our study was to explore which factor most affected the autonomic system dysfunction when PLMS occurred, with age and gender commonly considered to play a key role. In contrast to previous studies performed in small groups of patients [19,20], when we compared periods with and without PLMS in our sample, neither age and gender nor PLMS duration and density affected the degree of changes in parasympathetic and sympathetic tones during sleep. Furthermore, the magnitude of the HR response did not significantly differ according to gender and PLMS severity but was increased according to age and mean PLMS duration, with a larger tachycardia present when there was greater muscular activity. To explain the strong EMG effect in our sample, we can refer to a previous study that examined the changes in HRV and EEG spectra during PLMS, compared to changes in isolated leg movements and respiratory leg movements [37]. The authors found a greater sympathetic activation for PLMS compared to other movements; the markers of sympathetic activity such as the LF power correlated with EMG activity, and in particular with EMG magnitude.

These results may support the hypothesis that other factors, such as genetic profiles and individual vulnerability to PLMS, may confer susceptibility to RLS/PLMS [38] and autonomic dysfunction and cardiovascular disorders [39].

The strengths of this study include the following: a relatively large sample size of untreated patients with an equal presence of males and females, middle-aged and elderly cases, and moderate to severe RLS without neurological and cardiovascular disease; and a high number of examined PLMS to assess the magnitude of the cardiac response and the assessment of autonomic system in wakefulness, sleep and sleep stages. The most important limitation is the lack of data from healthy controls, which precludes firm conclusions. Comparison of the patterns of change in HR variables between RLS patients and heathy controls must be undertaken in future studies. Additionally, it should be mentioned that the interpretation of the LF/HF ratio is still debated by several authors [40].

To conclude, in our sample, the presence of PLMS did not contribute to increased sympathetic activity of basal conditions, and the magnitude of the cardiac PLMS-related response was independent of age, gender and PLMS severity, interval, periodicity and duration, with EMG activity being the major factor having an effect. Thus, we can say that the potential association between RLS-PLMS and cardiovascular risk is still hypothetical, and extensive longitudinal studies are needed to assess the real causality between RLS/PLMS autonomic activation and cardiovascular diseases.

## Figures and Tables

**Table 1:** Clinical and sleep data for all subjects and according to sex (means ± SD).

Variable	All (*n* = 50)	Women (*n* = 22)	Men (*n* = 28)	*p*
Age (y)	62.6 ± 11.1	63.1 ± 10.7	62.1 ± 11.6	0.77
Total sleep time (min)	394.6 ± 74.4	392.3 ± 59.2	396.3 ± 85.2	0.85
WASO (min)	99.2 ± 63.8	101.8 ± 56.7	97.2 ± 69.9	0.80
Awakenings (n)	25.8 ± 12.0	22.3 ± 9.2	28.5 ± 13.4	0.07
Sleep efficiency (%)	77.1 ± 12.9	77.4 ± 10.5	76.9 ± 14.8	0.89
Sleep latency (min)	16.7 ± 28.5	15.6 ± 24.3	17.7 ± 32.0	0.79
REM latency (min)	123.5 ± 61.8	128.7 ± 62.5	119.4 ± 62.1	0.60
Stage 1 (%)	12.1 ± 6.5	10.2 ± 3.3	13.6 ± 7.9	0.07
Stage 2 (%)	53.2 ± 7.8	53.3 ± 7.9	53.1 ± 7.9	0.96
Light sleep (%)	65.3 ± 8.8	63.4 ± 9.1	66.7 ± 8.5	0.19
SWS (%)	14.8 ± 6.7	16.5 ± 6.4	13.6 ± 6.7	0.13
REM (%)	19.8 ± 5.9	20.1 ± 6.0	19.7 ± 5.8	0.80
PLMS index (n/h)	45.7 ± 24.7	45.4 ± 22.1	45.9 ± 26.9	0.94
PLMS duration (s)	2.82 ± 0.95	3.03 ± 0.73	2.66 ± 1.08	0.17
PLMS IMI (s)	23.9 ± 4.5	23.7 ± 3.8	24.0 ± 5.0	0.83
PLMS PI (s)	0.70 ± 0.13	0.71 ± 0.11	0.70 ± 0.15	0.91

WASO: wakefulness after sleep onset; REM: rapid eye movement; light sleep (stage 1 + 2); SWS: slow wave sleep; PLMS: periodic leg movements; PLMS IMI: intermovement interval; PLMS PI: PLMS periodicity.

**Table 2:** Heart rate variability measures during wakefulness and sleep periods with and without PLMS (means ± SD).

	Without PLMS					With PLMS			
	Wake	Light Sleep	SWS	REM	Non-REM	Light Sleep	SWS	REM	Non-REM
HR (bpm)	74.5 ± 11.8	62.1 ± 10.2 +++	62.9 ± 9.5 +++	60.7 ± 8.8 +++	62.2 ± 9.2 +++	63.1 ± 9.3 +++	62.5 ± 7.9 +++	61.9 ± 9.0 +++	63.3 ± 9.1 +++
SDNN (ms)	42.1 ± 16.1	35.4 ± 15.1	30.0 ± 15.6 +++	48.7 ± 19.8	33.0 ± 14.0 +	56.8 ± 27.9 ***	49.3 ± 26.5 ***	66.2 ± 30.0 *++	55.5 ± 27.0 ***
pNN50 (%)	5.4 ± 8.8	10.2 ± 12.5	9.9 ± 14.7	9.3 ± 11.5	9.7 ± 12.1	13.3 ± 14.4 +	10.8 ± 14.0	14.1 ± 13.2	12.9 ± 14.0
rMSSD (ms)	23.5 ± 13.6	28.1 ± 14.3	28.2 ± 19.8	29.9 ± 17.5	27.5 ± 14.2	34.3 ± 19.3 ++	31.7 ± 19.5	37.7 ± 21.2 +	34.0 ± 18.9 +
Ptot (ms^2^)	794 ± 800	651 ± 603	487 ± 561 ++	1077 ± 967	583 ± 548	1841 ± 1780 ***++	1441 ± 1485 ***	2102 ± 2045 *++	1764 ± 1681 ***++
VLF (ms^2^)	390 ± 489	232 ± 250 +	141 ± 199 +++	530 ± 521	197 ± 223 +++	573 ± 512 ***	431 ± 444 ***	900 ± 816 ++	549 ± 477 ***
LF (ms^2^)	256 ± 219	259 ± 306	175 ± 260 ++	374 ± 364	226 ± 270	1069 ± 1153 ***+++	834 ± 931 ***++	931 ± 1182 *++	1018 ± 1084 ***+++
HF (ms^2^)	78 ± 85	130 ± 126	143 ± 192	110 ± 120	131 ± 118	165 ± 189 +	153 ± 214	192 ± 218	166 ± 191 +
LF/HF	5.67 ± 4.85	2.55 ± 1.81 +++	1.83 ± 1.73 +++	5.56 ± 5.04	2.38 ± 1.73 +++	8.89 ± 6.57 ***+	7.37 ± 5.88 ***	7.14 ± 4.85	8.51 ± 6.47 ***+

All HRV indices except HR were log-transformed before the statistical analyses. T-test: * *p* < 0.05, ** *p* < 0.01, *** *p* < 0.001 between periods with and without PLMS. ANOVA: + *p* < 0.05, ++ *p* < 0.01, +++ *p* < 0.001 between wakefulness and sleep stages.

**Table 3:** Heart rate variability indices during periods with PLMS according to sex and age (means ± SD).

	Light Sleep		Slow Wave Sleep		REM	
	Women	Men	Women	Men	Women	Men
HR (bpm)	64.1 ± 8.0	62.3 ± 10.3	64.4 ± 6.8	61.3 ± 8.4	62.2 ± 7.3	61.5 ± 11.7
SDNN (ms)	59.3 ± 24.3	54.8 ± 30.8	48.5 ± 26.5	49.8 ± 27.0	69.2 ± 29.7	61.7 ± 31.8
pNN50 (%)	14.6 ± 15.7	12.2 ± 13.4	10.0 ± 17.7	11.4 ± 11.4	16.0 ± 13.9	11.2 ± 12.3
rMSSD (ms)	35.9 ± 19.4	33.0 ± 19.4	30.5 ± 23.2	32.4 ± 17.2	40.3 ± 21.0	33.5 ± 22.2
Ptot (ms^2^)	1 908 ± 1646	1788 ± 1908	1413 ± 1646	1459 ± 1407	2412 ± 2265	1620 ± 1653
VLF (ms^2^)	629 ± 523	528 ± 509	456 ± 535	414 ± 385	1 040 ± 919	682 ± 610
LF (ms^2^)	1 053 ± 964	1082 ± 1300	758 ± 820	883 ± 1009	1062 ± 1303	726 ± 1001
HF (ms^2^)	187 ± 236	148 ± 145	175 ± 311	139 ± 124	226 ± 258	139 ± 132
LF/HF	8.89 ± 6.56	8.88 ± 6.70	7.56 ± 5.21	7.25 ± 6.38	6.84 ± 4.48	7.61 ± 5.63
	**Light sleep**		**Slow wave sleep**		**REM**	
	**<65 y (*n* = 25)**	**≥65 y (*n* = 25)**	**<65 y (*n* = 25)**	**≥65 y (*n* = 25)**	**<65 y (*n* = 25)**	**≥65 y (*n* = 25)**
HR (bpm)	63.6 ± 9.9	62.5 ± 8.9	63.2 ± 8.4	61.8 ± 7.3	63.8 ± 10.1	60.1 ± 7.9
SDNN (ms)	64.8 ± 32.0	48.7 ± 20.9	56.5 ± 30.7	41.8 ± 19.2	79.3 ± 34.7	54.2 ± 19.5
pNN50 (%)	15.7 ± 15.6	10.8 ± 12.8	14.7 ± 16.9	6.8 ± 8.9	16.4 ± 14.1	12.0 ± 12.7
rMSSD (ms)	38.3 ± 22.1	30.3 ± 15.4	36.1 ± 22.7	27.0 ± 14.6	40.0 ± 21.7	35.5 ± 21.5
Ptot (ms^2^)	2383 ± 2165	1298 ± 1084	1875 ± 1807	986 ± 883	2815 ± 2627	1449 ± 1056
VLF (ms^2^)	680 ± 603	465 ± 386	520 ± 511	337 ± 349	1052 ± 857	760 ± 787
LF (ms^2^)	1469 ± 1409	669 ± 630 *	1137 ± 1127	517 ± 528	1460 ± 1542	445 ± 304
HF (ms^2^)	203 ± 219	127 ± 149	196 ± 266	108 ± 134	223 ± 270	164 ± 164
LF/HF	9.30 ± 4.96	8.47 ± 7.95	6.82 ± 3.85	7.94 ± 7.52	8.55 ± 5.27	5.85 ± 4.24

All HRV indices except HR were log-transformed before the statistical analyses. * *p* < 0.05.

**Table 4:** Heart rate variability indices during periods with PLMS according to the PLM index and duration (mean ± SD).

	Light Sleep		Slow Wave Sleep		REM	
	<45/h (*n* = 25)	≥45/h (*n* = 25)	<45/h (*n* = 25)	≥45/h (*n* = 25)	<45/h (*n* = 25)	≥45/h (*n* = 25)
HR (bpm)	65.3 ± 10.6	60.8 ± 7.4	62.7 ± 9.1	62.3 ± 7.0	66.2 ± 9.0	58.0 ± 7.3 *
SDNN (ms)	53.9 ± 23.6	59.6 ± 31.9	48.8 ± 24.9	49.7 ± 28.3	60.7 ± 22.4	71.2 ± 35.9
pNN50 (%)	13.4 ± 14.2	13.2 ± 14.9	10.1 ± 11.8	11.4 ± 15.8	13.1 ± 11.5	15.0 ± 15.1
rMSSD (ms)	33.6 ± 19.1	35.0 ± 19.8	31.7 ± 19.7	31.6 ± 19.8	34.0 ± 18.2	41.0 ± 24.0
Ptot (ms^2^)	1572 ± 1220	2110 ± 2198	1324 ± 1165	1533 ± 1715	1554 ± 1005	2605 ± 2621
VLF (ms^2^)	495 ± 385	650 ± 613	466 ± 425	403 ± 466	674 ± 485	1107 ± 1010
LF (ms^2^)	871 ± 748	1267 ± 1440	685 ± 661	951 ± 1098	631 ± 568	1206 ± 1525
HF (ms^2^)	168 ± 170	162 ± 210	145 ± 164	159 ± 250	178 ± 153	205 ± 271
LF/HF	7.95 ± 5.18	9.82 ± 7.71	6.54 ± 4.84	8.01 ± 6.62	6.57 ± 4.96	7.66 ± 4.90
	**Light sleep**		**Slow wave sleep**		**REM**	
	**<2.7 s (*n* = 24)**	**≥2.7 s (*n* = 26)**	**<2.7 s (*n* = 24)**	**≥2.7 s (*n* = 26)**	**<2.7 s (*n* = 24)**	**≥2.7 s (*n* = 26)**
HR (bpm)	60.8 ± 9.3	65.1 ± 9.1	61.2 ± 7.3	63.9 ± 8.4	57.5 ± 9.1	64.8 ± 8.0
SDNN (ms)	48.7 ± 26.1	64.1 ± 28.0 *	42.6 ± 21.2	57.1 ± 30.3	53.5 ± 20.5	74.4 ± 32.9
pNN50 (%)	9.9 ± 11.5	16.4 ± 16.2	8.2 ± 10.0	13.8 ± 17.4	11.1 ± 12.2	16.0 ± 13.9
rMSSD (ms)	29.9 ± 16.4	38.4 ± 21.1	28.6 ± 15.9	35.2 ± 22.9	33.6 ± 21.4	40.3 ± 21.5
Ptot (ms^2^)	1409 ± 1648	2239 ± 1835 *	1055 ± 1110	1888 ± 1752	1429 ± 1090	2535 ± 2415
VLF (ms^2^)	468 ± 507	669 ± 508 *	325 ± 346	553 ± 518	791 ± 846	970 ± 820
LF (ms^2^)	811 ± 1071	1307 ± 1195 *	595 ± 740	1112 ± 1065	450 ± 317	1239 ± 1428
HF (ms^2^)	105 ± 93	221 ± 236	114 ± 135	198 ± 276	121 ± 135	238 ± 252
LF/HF	8.44 ± 6.50	9.30 ± 6.74	6.93 ± 5.21	7.87 ± 6.68	7.05 ± 4.39	7.20 ± 5.29

All HRV indices except HR were log-transformed before the statistical analyses. * *p* < 0.05.
